# The freshwater crabs of Macau, with the description of a new species of *Nanhaipotamon* Bott, 1968 and the redescription of *Nanhaipotamonwupingense* Cheng, Yang, Zhong & Li, 2003 (Crustacea, Decapoda, Potamidae)

**DOI:** 10.3897/zookeys.810.30726

**Published:** 2018-12-20

**Authors:** Chao Huang, Kai Chin Wong, Shane T. Ahyong

**Affiliations:** 1 Palaeontology, Geobiology and Earth Archives Research Centre, School of Biological, Earth and Environmental Sciences, University of New South Wales, Kensington, NSW 2052, Australia University of New South Wales Kensington Australia; 2 Australian Museum, 1 William St, Sydney NSW 2010, Australia Australian Museum Sydney Australia; 3 School of Life Sciences, Sun Yat-sen University, Guangzhou 510275, China Sun Yat-sen University Guangzhou China; 4 Macao Civic and Municipal Affairs Bureau, Macao SAR, China Macao Civic and Municipal Affairs Bureau Macao Macau

**Keywords:** Freshwater crabs, Gecarcinucidae, Macau, new species, Potamidae, systematics

## Abstract

Four species of freshwater crabs from three genera and two families (*Cantopotamonhengqinense* Huang, Ahyong & Shih, 2017, *Nanhaipotamonguangdongense* Dai, 1997, *Nanhaipotamonmacau***sp. n.**, and *Somanniathelphusazanklon* Ng & Dudgeon, 1992) are documented from Macau for the first time. One new species, *Nanhaipotamonmacau***sp. n.**, is described. The large flap on the male first gonopod terminal segment sets it apart from all other known congeners except *N.wupingense* Cheng, Yang, Zhong & Li, 2003, from Fujian. Characters of the carapace, male first gonopod and size, however, clearly differentiate these two species. Preliminary genetic studies also suggest that the two are not closely related. A neotype is designated for *N.wupingense*. The taxonomic status of *Nanhaipotamonguangdongense* is also discussed. Notes on the general biology and conservation status of these crabs are also included.

## Introduction

Best known as the gambling capital of the world, Macau (also known as Macao) has a total land area of only 30.8 km^2^ but a population of more than 650,000 people, making it one of the most densely populated regions in the world (Government of Macao Special Administrative Region Statistics and Census Service). Macau historically consists of the Macau Peninsula (bordered by Zhuhai to the north) and two islands: Taipa and Coloane. The two islands are now joined by Cotai, an area created by land reclamation in 2005.

**Figure 1.**
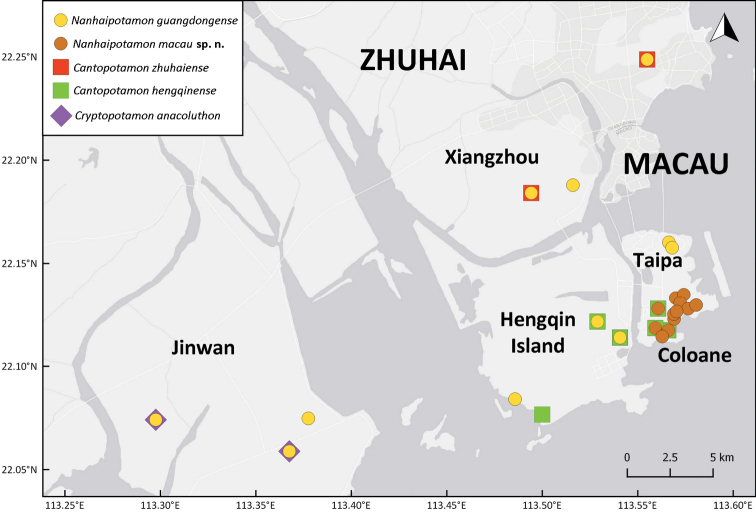
Localities of the sampling sites in and around Macau.

The freshwater crabs of Macau have not been scientifically documented to the best of our knowledge. General wetland faunal surveys from 2007 onwards have found freshwater crabs in Coloane resulting in a small collection kept in the Macao Civic and Municipal Affairs Bureau. Upon examination, it was found that these freshwater crab specimens contained three species, *Cantopotamonhengqinense* Huang, Ahyong & Shih, 2017, *Somanniathelphusazanklon* Ng & Dudgeon, 1992, and a new species of *Nanhaipotamon* Bott, 1968. This has led to more extensive surveys in 2018, covering 14 survey points (two in Taipa and 12 in Coloane; Fig. 1) focused exclusively on freshwater crabs. Three species of potamid crabs, *Cantopotamonhengqinense* Huang, Ahyong & Shih, 2017, *Nanhaipotamonguangdongense* Dai, 1997, and *Nanhaipotamonmacau* sp. n. and one species of gecarcinucid crab, *Somanniathelphusazanklon* Ng & Dudgeon, 1992, were found. *Nanhaipotamonmacau* sp. n. is very similar to the poorly known, *N.wupingense* Cheng, Yang, Zhong & Li, 2003, from Fujian Province. Unfortunately, the identity of *N.wupingense* is ambiguous because the type account is inadequate and the type material lost, requiring a neotype designation.

## Materials and methods

Specimens were collected by hand and preserved in 75% ethanol from 2007 onwards from South China. They are deposited in the Sun Yat-sen Museum of Biology, Sun Yat-sen University, Guangzhou, China (**SYSBM**); the Australian Museum, Sydney, Australia (**AM**); the Zoological Reference Collection of the Lee Kong Chian Natural History Museum, National University of Singapore, Singapore (**ZRC**); and the Macao Civic and Municipal Affairs Bureau (**IACM**). Measurements, in millimetres, are of the carapace width and length, respectively. Other abbreviations are as follows:

**G1** male first gonopod;

**G2** male second gonopod;

**CW** carapace width.

The terminology used primarily follows that of Dai (1999) and Davie et al. (2015). The Kimura 2-parameter (K2P) COI sequence distances (Kimura 1980) were calculated using MEGA6 (Tamura et al. 2013). H-T Shih kindly provided the COI sequence data of various species of *Nanhaipotamon* for use in this study (GenBank accession nos. MK226142-MK226145).

## Taxonomy

### Family Potamidae Ortmann, 1896

#### Subfamily Potamiscinae Bott, 1970

##### Genus *Cantopotamon* Huang, Ahyong & Shih, 2017

###### Cantopotamon hengqinense

Taxon classificationAnimaliaDecapodaPotamidae

Huang, Ahyong & Shih, 2017

Fig. 2C

Cantopotamon hengqinense Huang, Ahyong & Shih, 2017: 9, fig. 5.

####### Type material.

Holotype: SYSBM 001558, male (19.9 × 16.0 mm), Dahengqin Mountain (22.11N, 113.50E), Hengqin Island, Zhuhai City, Guangdong, China, small hillstream, under rocks, coll. C. Huang, February 2016. Paratypes: SYSBM 001559, 1 female (13.0 × 10.6 mm), same data as holotype. SYSBM 001560–001561, 2 males (15.5 × 12.4 mm, 13.2 × 10.7 mm), same data as holotype.

**Figure 2.**
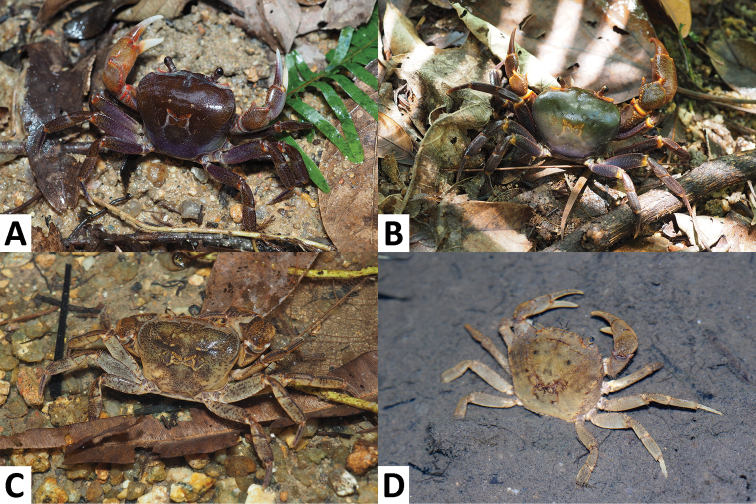
The freshwater crabs of Macau, colour in life. *Nanhaipotamonmacau* sp. n., male (29.0 × 24.2 mm), SYSBM 001654 (**A**); *Nanhaipotamonguangdongense* Dai, 1997, male (35.9 × 28.8 mm), SYSBM 001645 (**B**); *Cantopotamonhengqinense* Huang, Ahyong & Shih, 2017, male, specimen not collected (**C**); *Somanniathelphusazanklon* Ng & Dudgeon, 1992, photographed in Zhuhai, specimen not collected (**D**).

####### Other material examined.

China: IACM, 2 males (20.5 × 16.5 mm, 19.5 × 16.5 mm), Coloane (22.12N, 113.56E), Macau, small hillstream, under rocks, coll. K.C. Wong, November 2009. SYSBM 001640, 1 male (17.5 × 13.6 mm), Dahengqin Mountain, Hengqin Island, Zhuhai City, Guangdong, small hillstream, under rocks, coll. C. Huang, August 2017. SYSBM 001641–1644, 4 females (20.5 × 16.0 mm, 15.1 × 11.8 mm, 14.1 × 10.8 mm, 12.3 × 10.0 mm), same as SYSBM 001640. ZRC, 1 male (19.7 × 15.4 mm), Coloane, Macau, small hillstream, under rocks, coll. C. Huang, January 2018. ZRC, female (16.7 × 13.2 mm), same data as above. AM P101300, male (19.0 × 15.4 mm), Coloane, Macau, small hillstream, under rocks, coll. C. Huang, February 2018.

####### Distribution.

Hengqin Island, Zhuhai, Guangdong; Coloane, Macau.

####### Conservation status.

*Cantopotamonhengqinense* was previously only known from three hill streams in Dahengqin Mountain in Hengqin Island. This study found it to be present in another three hill streams in the neighbouring southwest corner of Coloane, Macau, which extends its extent of occurrence to 34.6 km^2^ (excluding sea area), area of occupancy to 11.7 km^2^ and number of locations to two. The populations in Hengqin and Macau are currently isolated from each other by a narrow strip of sea. Unlike the Hengqin population, whose habitat is threatened by urban development (Huang et al. 2017), the Macau population does not face serious imminent threat as all localities at which it was found are not currently open to development. Specimens from Macau are morphologically indistinguishable from those found in Hengqin Island. *Cantopotamonhengqinense* has not been found in Xiangzhou, Zhuhai to the north and Sanzao Island to the west despite considerable survey efforts during 2011–2018. In fact, no species of *Cantopotamon* are known from Sanzao Island, where instead *Cryptopotamonanacoluthon* (Kemp, 1918) is abundant. Given that the extent of occurrence and area of occupancy of *C.hengqinense* is much lower than 5, 000 km^2^ and 5000 km^2^, respectively, with fewer than five known locations and projected decline in habitat quality in Hengqin, the suggested corresponding conservation status of this species under IUCN Red List criteria remains as indicated by Huang et al. (2017), as Endangered B2(a)(b).

##### Genus *Nanhaipotamon* Bott, 1968

###### Nanhaipotamon macau sp. n.

Taxon classificationAnimaliaDecapodaPotamidae

http://zoobank.org/550E7796-5748-4522-9825-C2A6EDB3BD8B

**Figure 3.**
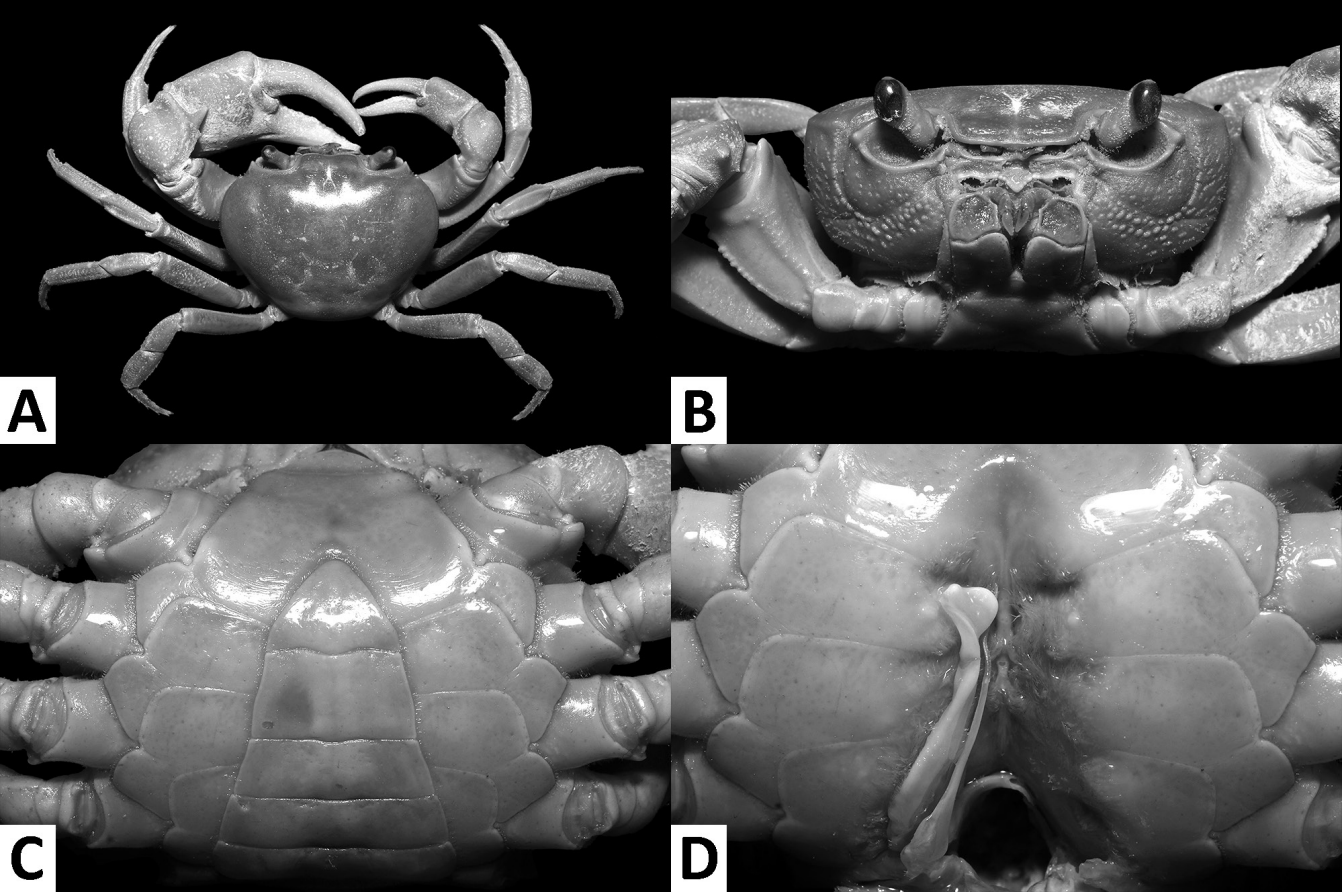
*Nanhaipotamonmacau* sp. n., male holotype (37.4 × 30.9 mm), SYSBM 001649 Dorsal habitus (**A**); cephalothorax, anterior view (**B**); anterior thoracic sternum and pleon, ventral view (**C**); sterno-pleonal cavity with right G1*in situ* (left G1 removed), ventral view (**D**).

**Figure 4.**
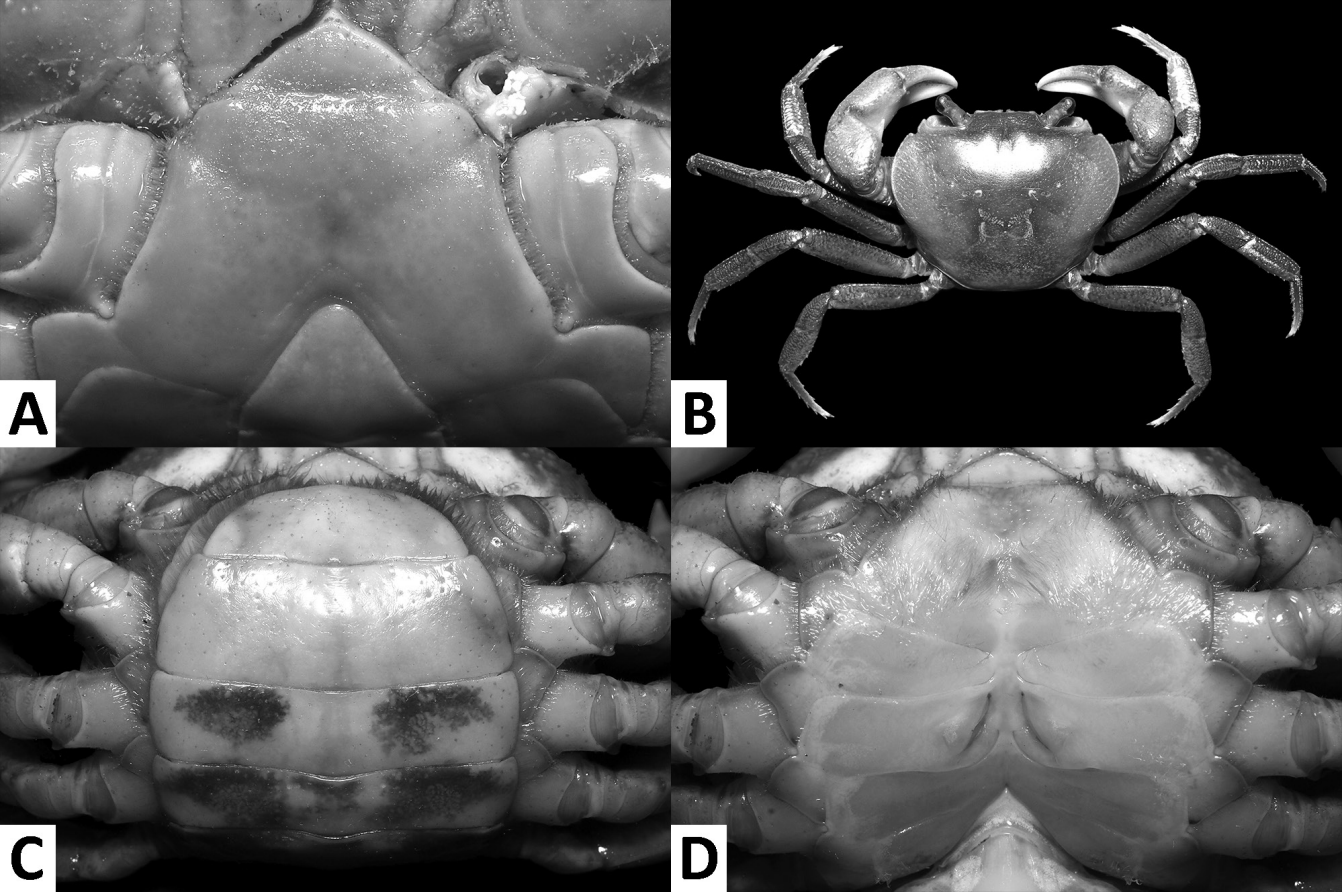
*Nanhaipotamonmacau* sp. n., male holotype (37.4 × 30.9 mm), SYSBM 001649 (**A**); female paratype (31.3 × 25.5 mm), SYSBM 001650 (**B–D**). Anterior thoracic sternum (**A**); dorsal habitus (**B**); pleon, ventral view (**C**); vulvae, ventral view (**D**).

**Figure 5.**
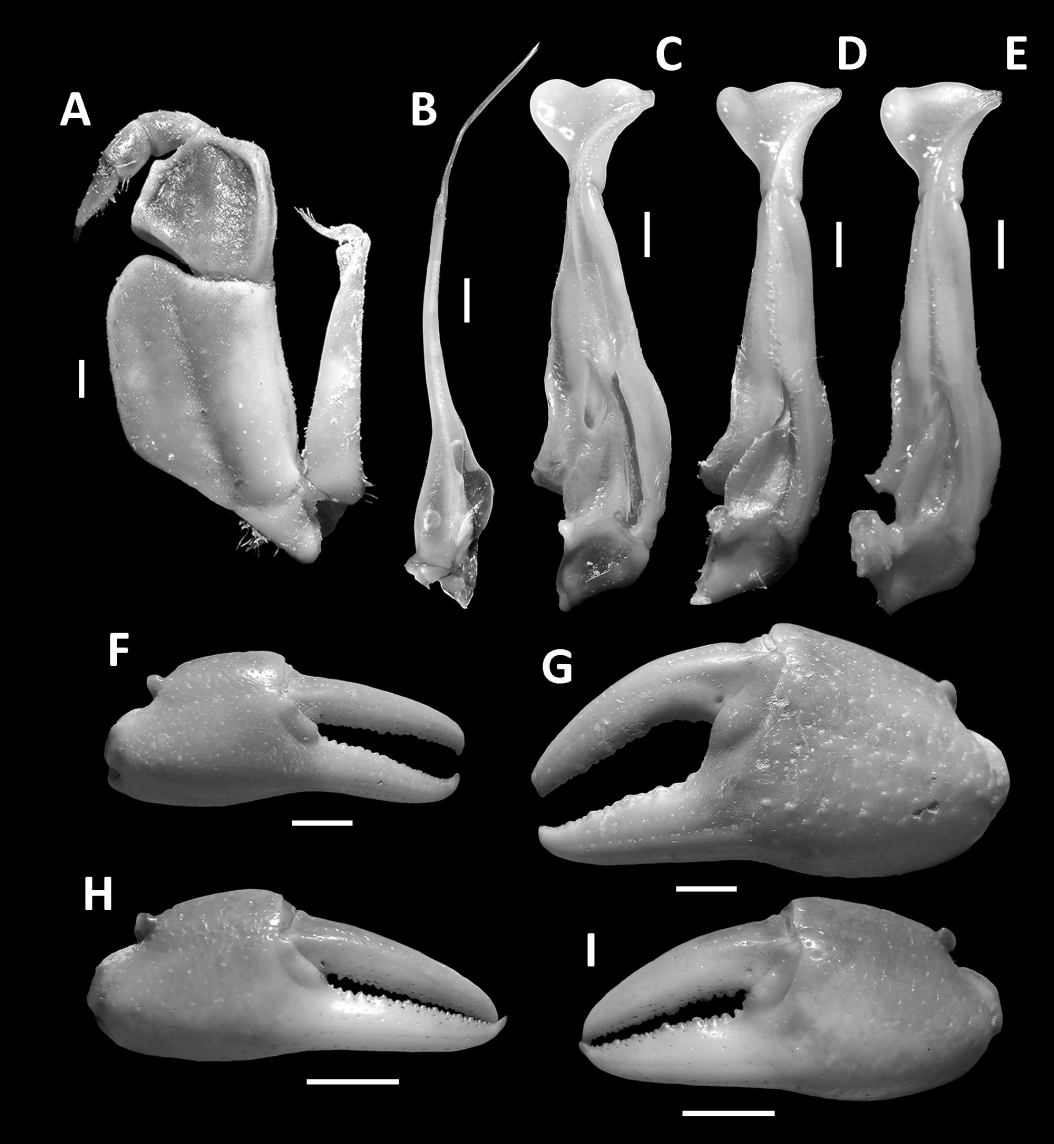
*Nanhaipotamonmacau* sp. n., male holotype (37.4 × 30.9 mm), SYSBM 001649 (**A–C, F, G**); male paratype (36.6 × 29.3 mm), SYSBM 001651 (**D**); male (35.3 × 28.8 mm), SYSBM 001652 (**E**); female paratype (31.3 × 25.5 mm), SYSBM 001650 (**H–I**). Left maxilliped 3 (**A**); left G2, ventral view (**B**); left G1, ventral view (**C–E**); minor cheliped (**F, H**); major cheliped (**G, I**). Scale bars: 1.0 mm (**A–E**); 5.0 mm (**F–I**).

**Figure 6.**
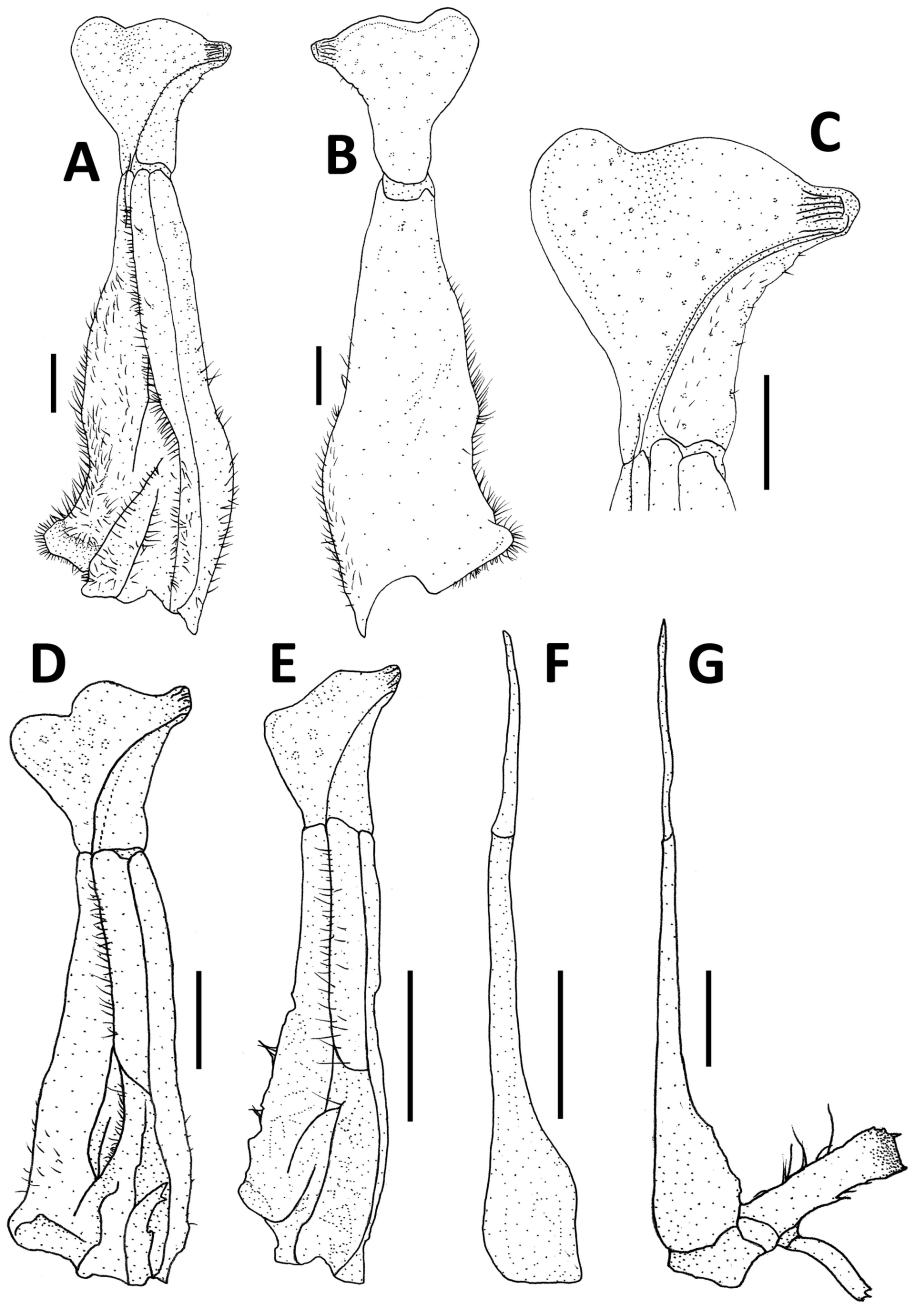
*Nanhaipotamonmacau* sp. n., male holotype (37.4 × 30.9 mm), SYSBM 001649 (**A–C**); *Nanhaipotamonwupingense* Cheng, Yang, Zhong & Li, 2003, male neotype (22.4 × 18.3 mm), JX 050563 (**D–F**); *Nanhaipotamonguangdongense* Dai, 1997, male, (30.9 × 24.8 mm), SYSBM 001646 (**G**). Left G1, ventral view (**A, D, E**); left G1, dorsal view (**B**); Left G1 terminal segment, ventral view (**C**); Left G2, ventral view (**F, G**). Scale bar: 1.0 mm.

Figs 2A , 3 , 4 , 5 , 6A–C

####### Type material.

Holotype: SYSBM 001649, male (37.4 × 30.9 mm), Coloane (22.12N, 113.56E), Macau, China, forest floor, coll. K.C. Wong, July 2010. Paratypes: SYSBM 001650, female (31.3 × 25.5 mm), Coloane, Macau, China, mud burrow adjacent to small hill stream, coll. C. Huang, February 2018. SYSBM 001651, male (36.6 × 29.3 mm), same data as above. IACM, 2 males (36.5 × 31.1 mm, 34.6 × 28.5 mm), Coloane, Macau, forest floor, coll. J. Z. Huang, July 2009. AM P101301, male (27.7 × 22.9 mm), same data as above. ZRC, male (22.3 × 19.0 mm) Coloane, Macau, China, under rock in small hillstream, coll. C. Huang, January 2018.

####### Other material examined.

Macau: SYSBM 001652-55, 4 males (35.3 × 28.8 mm, 32.1 × 26.5 mm, 29.0 × 24.2 mm, 28.9 × 24.1 mm), Coloane, in burrows adjacent to small hill stream, coll. C. Huang, February 2018.

####### Diagnosis.

Carapace broader than long, regions indistinct, dorsal surface convex, anterolateral region weakly rugose (Figs 3A, 4B); postorbital cristae sharp, laterally expanded, almost fused with epibranchial teeth and epigastric cristae (Figs 3A, 4B); external orbital angle sharply triangular, outer margin gently convex to almost straight, separated from anterolateral margin by conspicuous gap (Figs 3A, B, 4B); sub-orbital regions covered by sparse low granules, pterygostomial regions covered with short rows of a few rounded granules; sub-hepatic regions covered with lined striae (Fig. 3B); maxilliped III exopod reaching to proximal one-third of merus with short flagellum (Fig. 5A); female vulva ovate, medium-sized, positioned closely to one another (Fig. 4D); male pleon triangular, lateral margins almost straight (Fig. 3C); G1 slender, subterminal segment tapering distally, terminal segment large, distally expanded, distal margin laminar, apex blunt, directed outward (Figs 5C–E, 6A–C). G2 basal segment subovate (Fig. 5B).

####### Description.

Carapace broader than long, width about 1.2 × length (n = 6); regions indistinct, dorsal surface convex; surface generally smooth, pitted, anterolateral region weakly rugose (Figs 3A, 4B). Front deflexed, margin ridged in dorsal view (Figs 3A, 4B). Epigastric cristae low, separated by narrow gap (Figs 3A, 4B). Postorbital cristae sharp, laterally expanded, almost fused with epibranchial teeth and epigastric cristae (Figs 3A, 4B). Branchial regions slightly swollen (Figs 3A, B, 4B). Cervical groove shallow (Figs 3A, 4B). Mesogastric region slightly convex (Figs 3A, 4B). External orbital angle sharply triangular, outer margin gently convex to almost straight, separated from anterolateral margin by conspicuous gap (Figs 3A, B, 4B). Epibranchial tooth small, granular, indistinct (Figs 3A, B, 4B). Anterolateral margin cristate, lined with 20–23 granules, less distinct in some larger specimens; bent inward posteriorly (Figs 3A, 4B). Posterolateral surface with low, oblique striae, converging towards posterior carapace margin (Figs 3A, 4B). Orbits large; supraorbital, infraorbital margins cristate (Fig. 3B). Sub-orbital regions covered by sparse low granules, pterygostomial regions covered with short rows of a few rounded granules; sub-hepatic regions covered with lined striae (Fig. 3B). Epistome posterior margin narrow; median lobe broadly triangular, lateral margins slightly sinuous (Fig. 3B).

Maxilliped III merus about as wide as long; ischium width about 0.7 × length; merus subtrapezoidal, with median depression; ischium subtrapezoidal, with distinct median sulcus, mesial margin rounded. Exopod reaching to proximal one-third of merus; flagellum short (Fig. 5A).

Chelipeds (pereiopod I) unequal (Figs 3A, 5F–I); less inflated in females (Figs 4B, 5H, I). Merus trigonal in cross section; margins crenulated, dorsal-outer surface granulated. Carpus with sharp spine at inner-distal angle, spinule at base (Figs 3A, 4B). Major cheliped palm length about 1.2–1.4 × height in males (n = 5), 1.3 × in female (n = 1); dactylus 0.9–1.0 × palm length in males (n = 5), 0.9 × in female (n = 1) (Fig. 5G, I). Palm surface pitted, dorsal-outer surface granulated in larger males (Fig. 5G). Dactylus as long as pollex (Fig. 5F–I). Occlusal margin of fingers with irregular blunt teeth; slight gape when closed (Fig. 5F–I).

Ambulatory legs (pereiopods II–V) slender, setae short, very sparse (Figs 3A, 4B). Pereiopod III merus 0.6–0.7 × carapace length in males (n = 5), 0.6 × carapace length in female (n = 1) (Figs 3A, 4B). Pereiopods V propodus 2.1–2.2 × as long as broad in males (n = 5), 2.1 × as long as broad in female (n = 1), shorter than dactylus (Figs 3A, 4B).

Male thoracic sternum generally smooth; sternites I–IV narrow, width about 1.5 × length; sternites I, II forming triangular structure; sternites II, III fused, but demarcated by shallow transverse sulcus; sternites III, IV fused, demarcation inconspicuous (Fig. 4A). Male sterno-pleonal cavity reaching anteriorly beyond level of posterior articular condyle of cheliped coxa (Fig. 4A); deep median longitudinal groove between sternites VII, VIII (Fig. 3D). Male pleonal locking tubercle positioned at mid-length of sternite V (Fig. 3D). Female vulva ovate, medium-sized, not reaching the sutures of sternites V/VI or VI/VII, positioned closely to one another (Fig. 4D).

Male pleon triangular; somites III–VI progressively narrower, lateral margins almost straight; somite VI width 1.8–2.1 × length (n = 6); telson width 1.3–1.4 × length (n = 6); apex rounded (Fig. 3C). Female pleon broadly ovate (Fig. 4C).

G1 slender; in-situ, tip of terminal segment exceeding pleonal locking tubercle, almost reaching suture between thoracic sternites IV/V (Fig. 3D); subterminal segment length about 3.1 × length of terminal segment; subterminal segment tapering distally; terminal segment large, distally expanded, distal margin laminar, slightly sinuous to V-shaped, apex blunt, directed outward, orientation perpendicular to oblique to main axis of G1 (Figs 5C–E, 6A–C). G2 subterminal segment about 1.9 × length of flagelliform distal segment; exopod absent (Fig. 5B).

####### Etymology.

This species is named after the type locality, Macau; used as a noun in apposition.

####### Colour in life.

Variable, carapace and ambulatory legs dark brown to purple; chelipeds a combination of brown, orange and white (Fig. 2A).

####### Habitat.

*Nanhaipotamonmacau* sp. n. is a typical semi-terrestrial species that burrows in wet soil in the bank adjacent to hill streams. It was sympatric with *Cantopotamonhengqinense* at three localities (22.117N, 113.566E; 22.118N, 113.559E; 22.128N, 113.561E).

####### Distribution.

Coloane, Macau.

####### Remarks.

**Figure 7.**
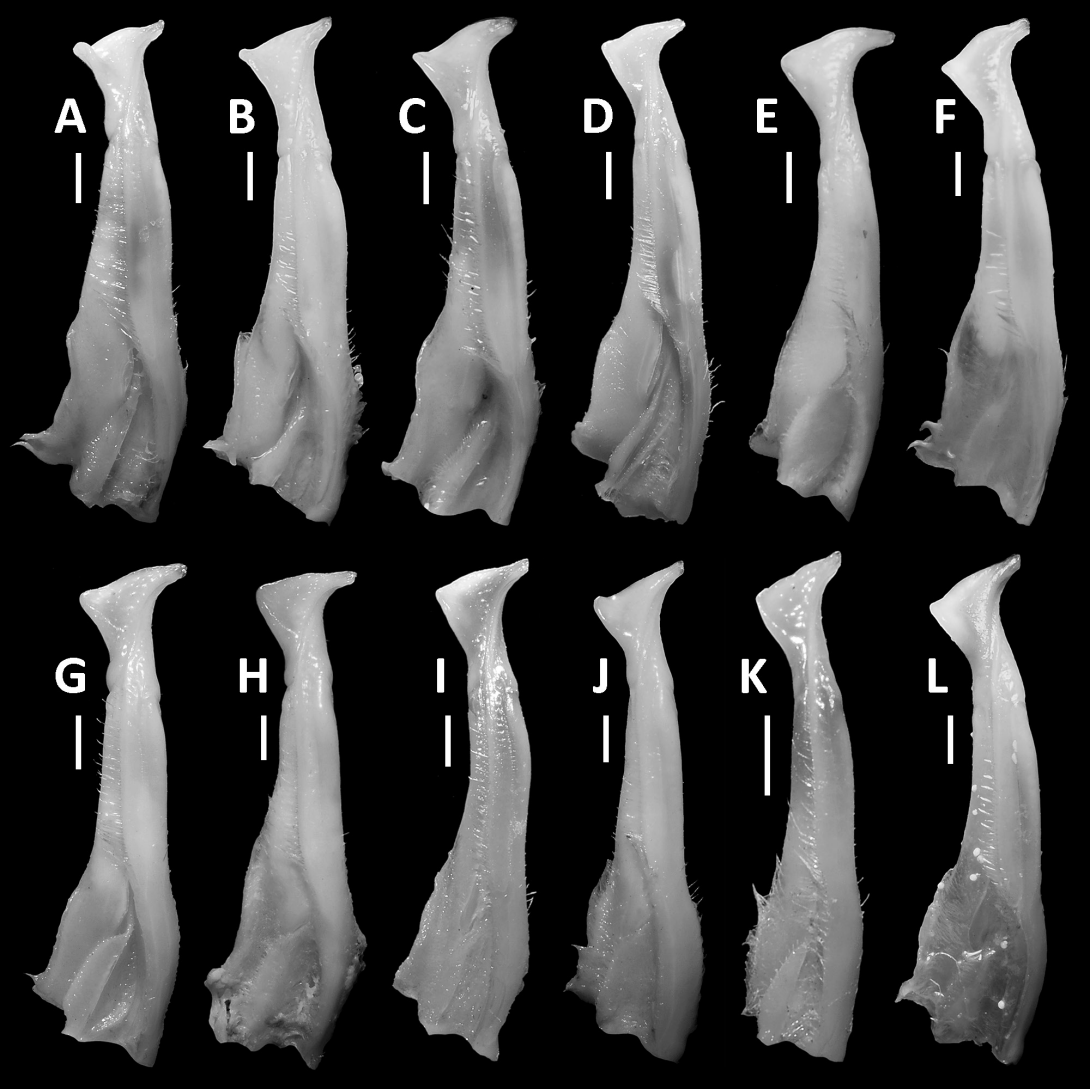
Comparison of G1 of *Nanhaipotamonguangdongense* Dai, 1997, from different localities. Male (38.4 × 31.5 mm), SYSBM 001141, Gujing, Jiangmen (**A**); male (40.8 × 32.2 mm), SYSBM 001142, Gujing, Jiangmen (**B**); male (36.7 × 29.4 mm), SYSBM 001143, Gujing, Jiangmen (**C**); male (35.4 × 29.4 mm), SYSBM 001177, Xiangzhou, Zhuhai (**D**); male (32.5 × 27.0 mm), SYSBM 001764, Xiangzhou, Zhuhai (**E**); male (40.1 × 32.7 mm), SYSBM 001758, Xiangzhou, Zhuhai (**F**); male (35.9 × 28.8 mm), SYSBM 001645, Coloane, Macau (**G**); male (45.5 × 37.0 mm), SYSBM 001656, Hengqin Island, Zhuhai (**H**); male (22.4 × 18.5 mm), SYSBM 001672, Jinwan, Zhuhai (**I**); male (33.4 × 26.5 mm), SYSBM 001017, Doumen, Zhuhai (**J**); male (36.9 × 30.4 mm), SYSBM 001657, Jinzhong Reservoir, Zhongshan (**K**); male (40.5 × 33.1 mm), SYSBM 001016, Qi’ao Island, Zhuhai (**L**). Scale bars: 1.0 mm.

As with many other species of *Nanhaipotamon*, *N.macau* sp. n. shows intraspecific variation in G1 morphology. In the terminal segment, the curves of the inner-distal and distal margins vary (Fig. 5C–E). The general shape and large size of the terminal segment, however, readily separates *N.macau* from other congeners in the Pearl River Delta Region, such as *N.guangdongense* Dai, 1997 (Fig. 7).

**Figure 8.**
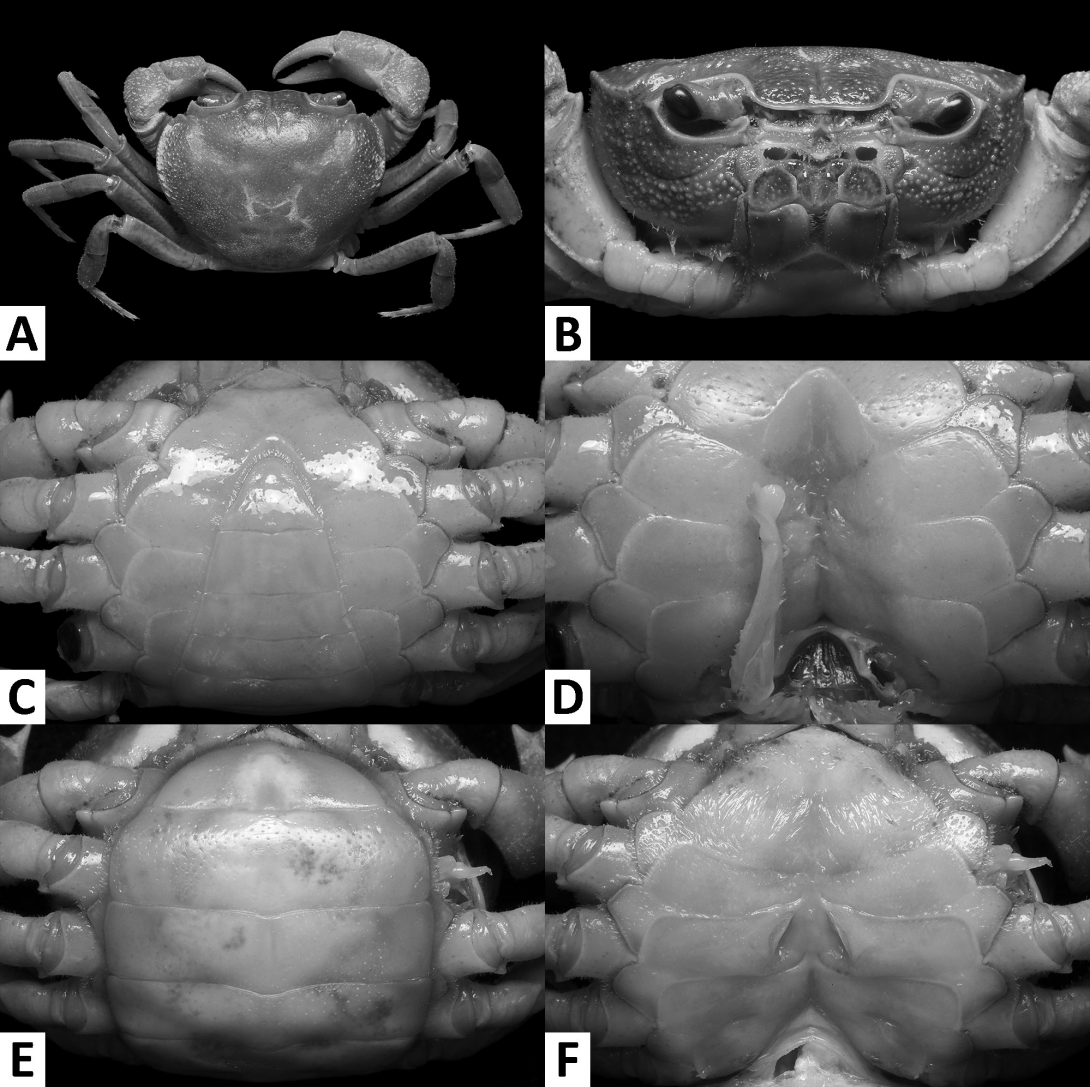
*Nanhaipotamonwupingense* Cheng, Yang, Zhong & Li, 2003, male neotype (22.4 × 18.3 mm), JX 050563 (**A–D**); female (25.3 × 20.8 mm), JX 050570 (**E–F**). Dorsal habitus (**A**); cephalothorax, anterior view (**B**); anterior thoracic sternum and pleon, ventral view (**C**); sterno-pleonal cavity with right G1*in situ* (left G1 removed), ventral view (**D**); pleon, ventral view (**E**); vulvae, ventral view (**F**).

*Nanhaipotamonwupingense* Cheng, Yang, Zhong & Li, 2003, from Fujian Province, is the only other known congener that also possesses such a large terminal segment. Based on the redscription of *N.wupingense* below, *N.macau* sp. n. differs by its larger maximum size (CW to 37.4 mm vs 27.5 mm in *N.wupingense*; Cheng et al. 2003); more inflated and less rugose branchial regions (compare Figs 3A, B, 8A, B); pterygostomial region granules larger, less numerous (compare Figs 3B, 8B); the G1 tip usually points laterally and the convex anterior margin next to the tip is often lower (Figs 5C–E, 6A–C) (tip points anterolaterally with higher adjacent convex margin, Fig. 6D; Cheng et al. 2003: fig. 7); G1 subterminal segment length about 3.0–3.2 × length of terminal segment in *N.macau* sp. n. (Figs 5C–E, 6A) (2.7 in the neotype of *N.wupingense*, see below, Fig. 6D). In keeping with their wide geographic separation, sequences of the COI barcoding region between *N.macau* sp. n. (SYSBM 001654; GenBank accession number MK226142) and *N.wupingense* (GenBank accession number: AB470511.1), courtesy of Hsi-Te Shih, shows a high (13.51%) Kimura 2-parameter (K2P) distance, corroborating their separate species status.

####### Conservation status.

*Nanhaipotamonmacau* sp. n. has an extremely restricted distribution with an extent of occurrence of only 5.3 km^2^ (excluding sea area) and an area of occupancy of around 3 km^2^. However, all 12 hill streams at which *N.macau* sp. n. was found are not currently open to urban development (one of these, Ka-Ho Reservoir Freshwater Wetland, is a protected area) and they seem to be locally abundant. We are unaware of any commercial harvesting of these crabs for human consumption or the aquarium trade. As such, no imminent threats to this species are apparent and it cannot be assigned to any level of threat according the IUCN Red List criteria. However, we emphasize the fragility of this species due to its highly restricted distribution; the habitat integrity of the hills of Coloane is paramount to this species’ survival.

###### Nanhaipotamon guangdongense

Taxon classificationAnimaliaDecapodaPotamidae

Dai, 1997

Figs 2B , 6G , 7

Nanhaipotamon guangdongense Dai, 1997: 229, fig. 9; Dai 1999: 121, pl. 8(1), fig. 60; Huang et al. 2012: 57, fig. 1A, 60; fig. 4, 61; fig. 5A–C.

####### Type material.

Holotype: AS-CB 05141, male (33.2 × 26.4 mm), Guangdong Province, China, gift from Sun Yat-Sen Medical College, no date [photographs examined].

####### Other material examined.

SYSU 001001, male (38.5 × 30.0 mm), Xiangzhou (22.25N, 113.57E), Zhuhai City, Guangdong, blue, mud hole next to small hillstream, coll. C. Huang, May 2012. SYSBM 001003, 1 male (36.2 × 28.4 mm), Xiangzhou, Zhuhai City, Guangdong, mud hole next to small hill stream, coll. C. Huang, February 2011. SYSBM 001004, 1 male (30.5 × 24.3 mm), Xiangzhou, Zhuhai City, Guangdong, mud hole next to small hill stream, coll. C. Huang, August 2012. SYSBM 001177, 1 male (35.4 × 29.4 mm), Xiangzhou, Zhuhai City, Guangdong, mud hole next to small hill stream, coll. C. Huang, May 2013. SYSBM 001178, 1 female, (35.9 × 29.4 mm), same data as above. SYSU 001758–001760, 3 males (40.1 × 32.7 mm, 36.0 × 29.8 mm, 30.2 × 25.1 mm), Xiangzhou, Zhuhai City, Guangdong, blue, mud hole next to small hillstream, coll. C. Huang, September 2018. SYSU 001761–001764, 4 males (42.1 × 33.5 mm, 40.5 × 32.4 mm, 38.1 × 32.0 mm, 32.5 × 27.0 mm), Xiangzhou, Zhuhai City, Guangdong, mud hole next to small hill stream, coll. C. Huang, September 2018. SYSBM 001141–001143, 3 males (38.4 × 31.5 mm, 40.8 × 32.2 mm, 36.7 × 29.4 mm), Gujing (22.36N, 113.12E), Jiangmen City, Guangdong, coll. local, August 2013. IACM, 2 males (39.5 × 31.5 mm, 25.2 × 21.1 mm), Taipa (22.16N, 113.58E), Macau, mud hole next to small hill stream, coll. K.C. Wong, March 2018. SYSBM 001645–001646, 2 males (35.9 × 28.8 mm, 30.9 × 24.8 mm), Taipa, Macau, mud hole next to small hill stream, coll. C. Huang, June 2018. SYSBM 001656, 1 male (45.5 × 37.0 mm), Dahengqin mountain (22.11N, 113.50E), Hengqin Island, Zhuhai City, Guangdong, in small hill stream pool, coll. C. Huang, August 2017. SYSBM 001672, 1 male (22.4 × 18.5 mm), Jinwan (22.08N, 113.35E), Zhuhai City, Guangdong, mud hole next to small hill stream, coll. C. Huang, June 2018. SYSBM 001673–001674, 2 females (31.7 × 25.7 mm, 25.9 × 20.8 mm), same data as above. SYSBM 001017–001019, 3 males (33.4 × 26.5 mm, 29.2 × 23.6 mm, 27.6 × 21.7 mm), Doumen (22.19N, 113.29E), Zhuhai City, Guangdong, coll. local, April 2013. SYSBM 001657–001658, 2 males (36.9 × 30.4 mm, 23.0 × 19.5 mm), Jinzhong Reservoir (22.48N, 113.38E), Zhongshan City, Guangdong, mud hole next to small hill stream, coll. C. Huang, January 2018. SYSBM 001659, 1 female (23.6 × 19.8), same data as above. SYSBM 001016, 1 male (40.5 × 33.1 mm), Qi’ao Island (22.43N, 113.66E), Zhuhai City, Guangdong, mud hole next to small hill stream, coll. C. Huang, May 2011. SYSBM 001023, 1 female (40.5 × 33.1 mm), same data as above. SYSBM 001750, 1 male (39.6 × 32.5 mm), Gudou Mountain (22.22N, 112.97E), Jiangmen City, Guangdong, coll. local, July 2018. SYSBM 001751, 1 male (35.1 × 26.0 mm), Xinhui (22.52N, 113.08E), Jiangmen City, Guangdong, coll. local, July 2018.

####### Colour in life.

Highly variable, even within the same population. Carapace and ambulatory legs dark brown to purple; chelipeds a combination of brown, orange and white (Fig. 2B). Blue variants are sometimes seen.

####### Distribution.

Guangdong: Zhuhai, Zhongshan, Jiangmen; Macau: Taipa.

####### Remarks.

*Nanhaipotamonguangdongense* has been found at only one locality in Macau (Tai Tam Hill, Taipa). One specimen (SYSBM 001646) has exopods on the G2 on both sides, the first such report for a freshwater crab (Fig. 6G). The G2 exopod is likely the result of a developmental abnormality and is an extremely rare occurrence (Gordon, 1963). The majority of brachyurans lack the male G2 exopod although it is being increasingly recognized as a normal feature among many pinnotherid crabs (Ahyong et al. 2012; Ng and Ho 2016).

Little was previously known about *N.guangdongense* as it was described from a single specimen without a precise locality. Attempts to sequence the DNA of *N.guangdongense* were unsuccessful, probably because of formalin fixation, compounding the problem of its identification (Shih et al. 2011). Huang et al. (2012) reported *N.guangdongense* from Zhuhai. Small differences in the G1 morphology, however, suggest the holotype of *N.guangdongense* was probably collected from another locality (Peter Ng pers. comm.). More recent collection efforts in Guangdong have found this species at multiple locations in Zhuhai, Zhongshan, and Jiangmen. Specimens from Gujing, Jiangmen (Fig. 7A–C) most closely resemble the holotype in G1 morphology suggesting that the holotype was probably collected from that area.

Normal and blue coloured *Nanhaipotamon* were sympatric at a locality in Xiangzhou, Zhuhai. *Nanhaipotamonzhuhaiense* Huang, Huang & Ng, 2012 was described based on only three blue specimens that had a distinctive G1 that pointed laterally and not anterolaterally as seen in the normal coloured comparative specimens. More recent collections from Xiangzhou, Zhuhai, however, have found a normal coloured specimen that has a laterally pointing G1 (Fig. 7E) and also a blue specimen that has an anterolaterally pointing G1 (Fig. 7F). Therefore, the colouration of the crab does not always correspond to a particular gonopod morphology. Specimens of intermediate G1 morphology have also been collected, while one uncollected female specimen was observed to be of intermediate colour. Furthermore, the COI K2P distances between the blue specimens SYSBM 001001 (GenBank no: MK226143), SYSBM001249 (GenBank no: MK226144) and the normal coloured specimen SYSBM 001015 (GenBank no: MK226145) are 1.23% and 0.77% respectively, which is of intraspecific level among closely related congeners. This new evidence strongly suggests that the normal and blue coloured crabs are different colour phases of the same species, but the G1 morphological differences between different specimens remains to be further studied. This seems to be a similar case to that of *N.hepingense* Dai, 1977, and *N.pinghense* Dai, 1977 (see Shih et al. 2011; Huang et al. 2012). Given that we are unable to confidently separate *N.zhuhaiense* from *N.guangdongense*, we regard them as probably conspecific, but refrain from making formal taxonomic changes until further detailed comparisons can be completed.

The G1 of specimens of *N.guangdongense* from different localities varies (Fig. 7). It is becoming increasingly evident that intraspecific variation of gonopodal morphology in some species of *Nanhaipotamon* is wider than previously recognized, while external differences are often hard to detect between species, making the taxonomy of this genus problematic (Figs 5C–E, 7; Huang et al., 2012: fig. 5; unpublished data). Clearly, there is need for a revision of this genus. To avoid compounding the problem in the future, we strongly recommend that new species of *Nanhaipotamon* should only be described when a large series of specimens is available to account for intraspecific variation.

####### Conservation status.

*Nanhaipotamonguangdongense* was previously assessed as Data Deficient, being known from one unspecified location in Guangdong (Cumberlidge 2008). This species is sometimes collected for food and for the pet trade, though we are uncertain as to the extent. Nevertheless, this species has been found in many locations with a wider range than previously thought, having an extent of occurrence of around 2,400 km^2^ (excluding sea area) and an area of occupancy of around 1,600 km^2^. As such, we suggest the conservation status of this species under IUCN criteria would be more appropriate as Least Concern (LC). Nevertheless, *N.guangdongense* is quite rare in Macau, being found in only one location in the Ecological Pond of Grand Taipa, and thus may warrant local conservation attention.

###### Nanhaipotamon wupingense

Taxon classificationAnimaliaDecapodaPotamidae

Cheng, Yang, Zhong & Li, 2003

Figs 6D–F , 8

Nanhaipotamon wupingense Cheng, Yang, Zhong & Li, 2003: 678, figs 1–8.

####### Type material.

Neotype: JX 050563, male (22.4 × 18.3 mm), Xiaba (24.89N, 116.05E), Wuping county, Longyan City, Fujian Province, China, coll. X. M. Zhou, May 2007.

####### Other material examined.

JX 050564, JX 050566, JX 050568–050569, 4 males (16.2 × 13.2 mm, 15.5 × 12.6 mm, 13.0 × 10.9 mm, 14.0 × 11.5 mm), same data as neotype. JX 050565, JX 050567, JX 050570–050576, 9 females (16.1 × 13.2 mm, 13.0 × 10.5 mm, 25.3 × 20.8 mm, 23.4 × 19.5 mm, 24.9 × 20.3 mm, 21.6 × 17.6 mm, 16.9 × 13.7 mm, 14.8 × 11.7 mm, 15.2 × 12.4 mm), same data as neotype.

####### Description.

Carapace broader than long, width about 1.2 × length (n = 14); regions indistinct, dorsal surface convex; surface generally smooth, pitted, anterolateral region slightly rugose (Fig. 8A). Front deflexed, margin ridged in dorsal view (Fig. 8A). Epigastric cristae low, separated by narrow gap (Fig. 8A). Postorbital cristae sharp, laterally expanded, almost confluent with epibranchial teeth and epigastric cristae (Fig. 8A). Branchial regions slightly swollen (Fig. 8A, B). Cervical groove shallow (Fig. 8A). Mesogastric region slightly convex (Fig. 8A). External orbital angle sharply triangular, outer margin gently convex, separated from anterolateral margin by conspicuous gap (Fig. 8A, B). Epibranchial tooth small, granular, indistinct (Fig. 8A, B). Anterolateral margin cristate, lined with 17–20 granules, less distinct in some larger specimens; curved inward posteriorly (Fig. 8A). Posterolateral surface with low, oblique striae, converging towards posterior carapace margin (Fig. 8A). Orbits large; supraorbital, infraorbital margins cristate (Fig. 8B). Sub-orbital, pterygostomial and sub-hepatic regions covered with numerous rounded granules (Fig. 8B). Epistome posterior margin narrow; median lobe broadly triangular, lateral margins slightly sinuous (Fig. 8B).

Maxilliped III merus about as wide as long; ischium width about 0.7 × length; merus subtrapezoidal, with median depression; ischium subtrapezoidal, with distinct median sulcus, mesial margin rounded. Exopod reaching to proximal one-third of merus; flagellum short.

Chelipeds (pereiopod I) unequal (Fig. 8A); less inflated in females. Merus trigonal in cross section; margins crenulated, dorsal-outer surface granulated (Fig. 8A). Carpus with sharp spine at inner-distal angle, spinule at base (Fig. 8A). Major cheliped palm length about 1.3 × height (n = 1) in males, 1.3–1.4 × height (n = 4) in females; dactylus about 1.0 × palm length (n = 1) in males, 1.0–1.1 × palm length (n = 4) in females. Palm surface pitted. Dactylus as long as pollex (Cheng et al. 2003: fig. 1). Occlusal margin of fingers with irregular blunt teeth; slight gape when closed.

Ambulatory legs (pereiopods II–V) slender, setae short, very sparse (Fig. 8A). Pereiopod III merus 0.6 × carapace length (n = 3) in males, 0.6–0.7 × carapace length (n = 7) in females (Fig. 8A). Pereiopods V propodus 2.3–2.4 × as long as broad in males (n = 3), 2.3–2.4 × as long as broad in females (n = 5), shorter than dactylus (Fig. 8A).

Male thoracic sternum generally smooth; anterior thoracic sternum (sternites I–IV) narrow, width about 1.5 × length; sternites I, II forming triangular structure; demarcation between sternites II, III complete; sternites III, IV fused with vestigial median suture (Fig. 8C). Male sterno-pleonal cavity reaching anteriorly beyond level of posterior articular condyle of cheliped coxa (Fig. 8C); deep median longitudinal groove between sternites VII, VIII (Fig. 8D). Male pleonal locking tubercle positioned at mid-length of sternite V (Fig. 8D). Female vulva ovate, not reaching the sutures of sternites V/VI or VI/VII, positioned closely to one another (Fig. 8F).

Male pleon triangular, lateral margins almost straight; somites III–VI progressively narrower; somite VI width 2.1–2.2 × length (n=2); telson width 1.2–1.3 × length (n=2); apex rounded (Fig. 8C). Female pleon broadly ovate (Fig. 8E).

G1 slender; in-situ, tip of terminal segment exceeding pleonal locking tubercle, reaching suture between thoracic sternites IV/V (Fig. 8D, G1 not flat against the body); subterminal segment length about 2.7 × length of terminal segment; subterminal segment tapering posteriorly; terminal segment large, distally expanded, anterior margin laminar, convex anterior margin next to the tip high, tip blunt (Fig. 6D, E). G2 subterminal segment about 2.1 × length of flagelliform distal segment; exopod absent (6F).

####### Distribution.

Currently only known from Xiaba, Wuping County, Longyan City, Fujian.

####### Remarks.

The original description of *Nanhaipotamonwupingense* is brief and minimally illustrated (Cheng et al. 2003), neither describing nor figuring details of the carapace physiognomy and pterygostomial ornamentation, which are diagnostic differences between *N.wupingense* and *N.macau* sp. n. Although the gonopods of *N.wupingense* were described and figured, and were distinctive at the time of original description, they are similar to that to the newly discovered *N.macau* sp. n. As such the type account of *N.wupingense* could apply equally to *N.macau* sp. n. Unfortunately, the type material of *N.wupingense* is now lost: according to the first author of *N.wupingense*, the type material of *N.wupingense*, which was originally deposited in Fujian Research Institute of Parasite Disease, Fuzhou, Fujian Province, was lost during relocation (YZ Cheng, pers. comm.). Therefore, in order to fix the identity of *N.wupingense* and allow adequate characterization of both species, we hereby designate a neotype for *N.wupingense* in accordance to ICZN (1999: art. 75.3). The neotype of *N.wupingense* (male, 22.4 × 18.3 mm, JX 050563) and other examined specimens of the species were collected from the original type locality. The neotype G1 corresponds well to that originally described and figured for *N.wupingense*, although we note some minor differences in morphometrics compared to the original type description (Cheng et al. 2003). The G1 subterminal/terminal segment length ratio of *N.wupingense* is 3.0 according to Cheng et al. (2003), but, we measure the ratio at 2.7 in the neotype (Fig. 6D) and 2.6 based on the illustration of the G1 of the holotype (Cheng et al. 2003: fig. 7). The G2 subterminal/terminal segment length ratio of *N.wupingense* is inconsistently recorded in Cheng et al. (2003): 1.8 in the Chinese description, erroneously as 2.7 in the English abstract, and 2.0 if based on the figure of the holotype G2 (Cheng et. al. 2003: fig. 6). This ratio could not be measured for the neotype as the G2 terminal segment broke off inside the G1 during dissection, although the ratio in JX 050564, a sub-adult, is 2.2 (Fig. 6F). The G1 is not fully developed in this specimen (Fig. 6E).

### Family Gecarcinucidae Rathbun, 1904

#### Genus *Somanniathelphusa* Bott, 1968

##### Somanniathelphusa zanklon

Taxon classificationAnimaliaDecapodaGecarcinucidae

Ng & Dudgeon, 1992

Figs 2D , 9

Somanniathelphusa zanklon Ng & Dudgeon, 1992: figs 11–13.
Parathelphusa sinensis : Doflein 1902: 662.
Parathelphusa (Parathelphusa) sinensis : Gee 1925: 159; Wu 1934: 339.
Somanniathelphusa sinensis sinensis : Bott 1968b: 409, figs 11, 12, 30; Bott 1970a: 338;
Bott 1970b: 111, pl. 20, figs 42–44; Ng, 1988: 105.
Somanniathelphusa sinensis : Dai 1999: 67, fig. 29, pl. 2.

###### Material examined.

SYSBM 101001, 1 male (27.2 × 23.1 mm), Nanping (22.19N, 113.5E), Zhuhai City, Guangdong, reservoir, coll. C. Huang, April 2015. SYSBM 101002–101003, 2 males (38.8 × 31.9 mm, 30.4 × 25.6 mm), Jinding (22.38N, 113.54E), Zhuhai City, Guangdong, coll. local, May 2014. SYSBM 101004–101005, 2 males (42.0 × 33.5 mm, 34.2 × 28.1 mm), Sun Yat-sen University (23.10N, 113.30E), Guangzhou City, Guangdong, fish pond, coll. C. Huang, June 2013. SYSBM 101006, 1 female (37.1 × 29.5 mm), same data as above. SYSBM 101007, 1 male (30.6 × 24.5 mm), Coloane (22.12N, 113.56E), Macau, reservoir, coll. K.C. Wong, July 2008. SYSBM 101008, 1 male (28.2 × 22.5 mm), Coloane, Macau, reservoir, coll. K.C. Wong, July 2009. IACM, 1 male (24.2 × 19.8 mm), Coloane, Macau, reservoir, coll. K.C. Wong, February 2013. SYSBM 101009–101010, 2 males (19.5 × 16.8 mm, 19.2 × 16.4 mm), Shenzhen City (22.6N, 114.0E), Guangdong, coll. local, August 2015. SYSBM 101011, 1 female (19.6 × 16.4 mm), same data as above. SYSBM 101015–101016, 2 males (35.7 × 28.4 mm, 37.2 × 29.9 mm), Sihui City (23.12N, 113.56E), Guangdong, coll. local, August 2013. SYSBM 101017, 1 female (31.6 × 25.9 mm), same data as above. SYSBM 101018–101020, 3 males (38.2 × 30.8 mm, 35.4 × 28.1 mm, 28.3 × 21.3 mm), Renhua, Shaoguan City, Guangdong, coll. local, August 2013. SYSBM 101021, 1 male (31.5 × 26.6 mm), Lianhua Mountain, Shanwei City, Guangdong, coll. local, October 2013. SYSBM 101022, 1 female (27.3 × 23.2 mm), same data as above. SYSBM 101030, 1 male (39.4 × 33.0 mm), Heyuan City, Guangdong, coll. Z.C. Zhou, January 2014. SYSBM 101031, 1 female (32.4 × 27.3 mm), same data as above. SYSBM 101032–101035, 4 males (35.0 × 28.3 mm, 32.7 × 26.9 mm, 30.8 × 25.0 mm, 25.9 × 21.7 mm), Raoping, Chaozhou City, Guangdong, coll. Z.C. Zhou, January 2014. SYSBM 101036, 1 female (24.6 × 20.8 mm), same data as above. SYSBM 101037–101040, 4 males (26.3 × 20.4 mm, 30.9 × 25.8 mm, 26.6 × 23.0 mm, 27.0 × 22.5 mm), Wenzhou City, Zhejiang, coll. local, October 2013. SYSBM 101041, 1 female (29.8 × 24.6 mm), same data as above.

###### Colour in life.

Generally brown overall; larger individuals may have dark markings near the cardiac region (Fig. 2D).

###### Distribution.

Coloane, Macau; Guangdong: Guangzhou City, Shenzhen City, Zhuhai City, Sihui City, Shaoguan City, Shanwei City, Heyuan City, Chaozhou City; Zhejiang: Wenzhou City.

###### Remarks.

**Figure 9.**
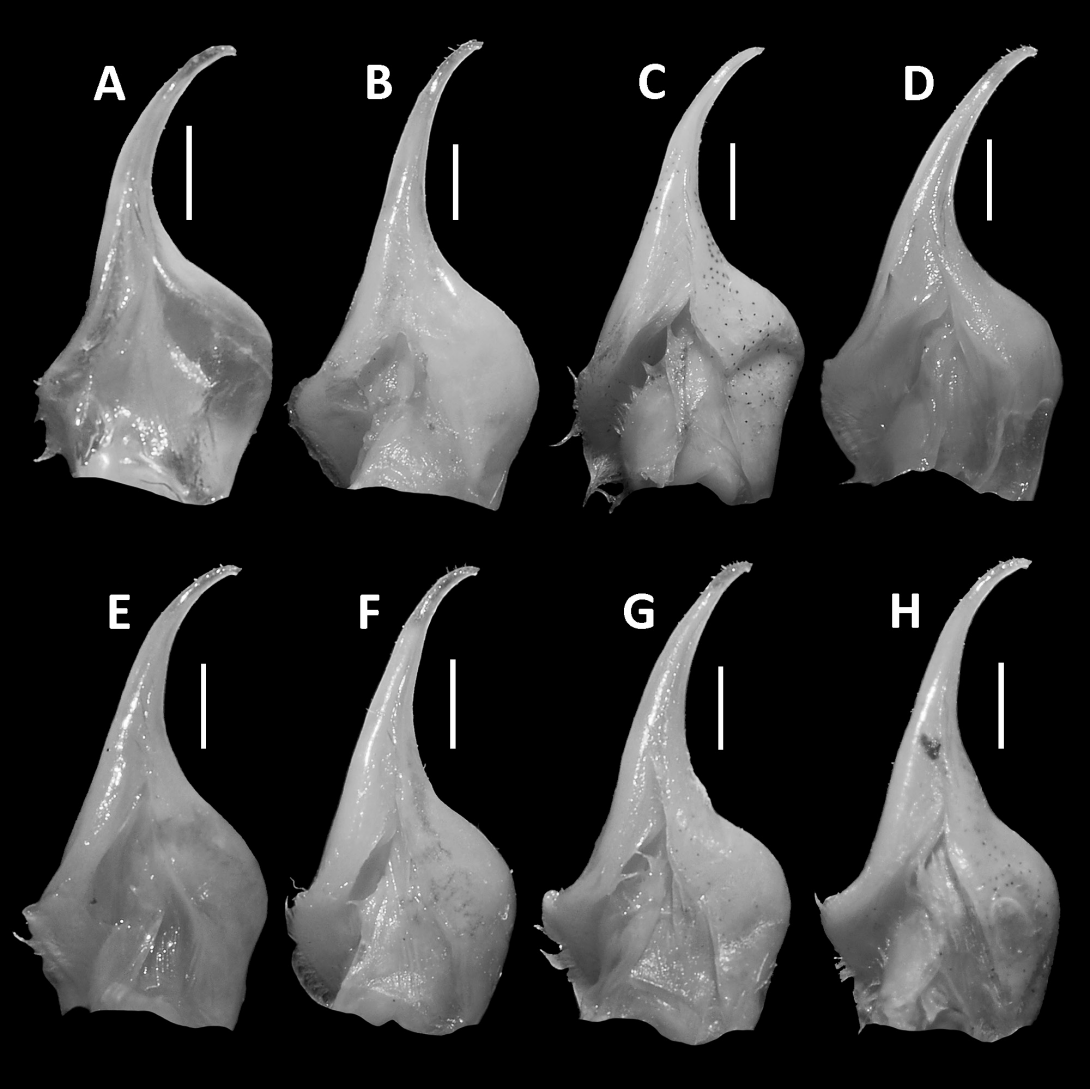
Comparison of G1 of *Somanniathelphusazanklon* Ng & Dudgeon, 1992, from different localities. Male (30.6 × 24.5 mm), SYSBM 101007, Coloane, Macau (**A**); male (38.8 × 31.9 mm), SYSBM 101002, Jinding, Zhuhai City, Guangdong (**B**); male (42.0 × 33.5 mm), SYSBM 101004, Sun Yat-sen University, Guangzhou City, Guangdong (**C**); male (35.7 × 28.4 mm), SYSBM 101015, Sihui City, Guangdong (**D**); male (28.3 × 21.3 mm), SYSBM 101020, Renhua, Shaoguan City, Guangdong (**E**); male (31.5 × 26.6 mm), SYSBM 101021, Lianhua Mountain, Shanwei City, Guangdong (**F**); male (35.0 × 28.3 mm), SYSBM 101032, Raoping, Chaozhou City, Guangdong (**G**); male (26.3 × 20.4 mm), SYSBM 101037, Wenzhou City, Zhejiang (**H**). Scale bars: 1.0 mm.

Ng and Dudgeon (1992) showed former records of *Somanniathelphusasinensis* (H. Milne Edwards, 1853) from southern China (Bott 1968, 1970; Dai 1999) to represent a new species, *S.zanklon.*Shih et al. (2007) included *S.zanklon* from Hong Kong and Guangdong (Dongguan City and Nanhai City) and showed that specimens of *Somanniathelphusa* from eastern Guangdong, Fujian and west-central Taiwan were all very closely related genetically, probably even conspecific. Specimens examined here from different localities were all very similar morphologically (Fig. 9), with those from Zhejiang Province having a slightly longer G1 distal part (Fig. 9H), though the overall shape is very much like the others. We tentatively treat all of these as the same species pending a full revision. Interestingly, Dai (1999) also reported the genus from Zhejiang, but none of the species of *Somanniathelphusa* in her monograph lists Zhejiang among its localities. The Chinese *Somanniathelphusa* are particularly problematic as many species look identical externally and were described based on minute differences that we find difficult to detect. Furthermore, being lowland species, they are readily dispersed by floods and are commonly found in aquaculture ponds where their newly hatched crablings are easily translocated. Preliminary genetic evidence suggests that the species diversity of *Somanniathelphusa* may have been overestimated (Shih et al. 2007).

###### Conservation status.

*Somanniathelphusazanklon* is currently assessed as Endangered (Esser and Cumberlidge 2008) as it was known from fewer than five locations in Hong Kong with a extant of occurrence less than 5,000 km^2^, with degrading habitat quality. Our study, however, finds this species to have a widespread occurrence in south-east China with an area of occupancy estimated at over 120,000 km^2^. Though habitat quality in some of these locations is declining, this resilient lowland species seems to be able to thrive in most water bodies that are not heavily polluted. As such, we find that *S.zanklon* does not satisfy any IUCN Red List threat categories and thus we suggest Least Concern would be a more appropriate determination at present. In Macau, all known occurrences of *S.zanklon* are from Hac-Sa Reservoir, Coloane, although it most likely also occurs in other water bodies in Coloane. There was an unconfirmed sighting of *S.zanklon* in Taipa a few years ago by the second author, though more recent surveys have failed to locate any specimens. There are three large water bodies on the Macau peninsula, of which only one reservoir on the east, next to Parque Municipal do Monte da Guia, which sources freshwater from the mainland, is suitable for lowland freshwater crabs. Although we did not survey this reservoir, it very likely also holds *S.zanklon* as this species was found in one of its water source reservoirs in Zhuhai. The other two water bodies, Sai Van Lake and Nam Van Lake, were once bays that have mostly been artificially closed off by landfill and currently hold sea water.

## Supplementary Material

XML Treatment for Cantopotamon hengqinense

XML Treatment for Nanhaipotamon macau

XML Treatment for Nanhaipotamon guangdongense

XML Treatment for Nanhaipotamon wupingense

XML Treatment for Somanniathelphusa zanklon
